# Effectiveness of a brief motivational intervention in the management of risky alcohol use in primary care: ALCO-AP20 study protocol

**DOI:** 10.3389/fmed.2022.1008832

**Published:** 2023-01-13

**Authors:** Esperanza Romero-Rodríguez, Celia Pérula-Jiménez, Sara Fernández-López, Gregorio Cabello-Gracia, José Ángel Fernández-García, Luis Ángel Pérula-de Torres, Ana Roldán-Villalobos, Fernando Leiva-Cepas, Rodrigo Fernández-Márquez, Juan Manuel Parras-Rejano, ALCO-AP-20 Collaborative Study Group

**Affiliations:** ^1^Reina Sofia University Hospital, Maimonides Biomedical Research Institute of Cordoba (IMIBIC), University of Córdoba, Córdoba, Spain; ^2^Multiprofessional Teaching Unit of Family and Community Care of Córdoba, Health District of Córdoba and Guadalquivir, Córdoba, Spain; ^3^Andalusian Health Service, Carlos Castilla del Pino Health Center, Córdoba, Spain; ^4^Andalusian Health Service, Montoro Health Center, Córdoba, Spain; ^5^Andalusian Health Service, Peñarroya Health Center, Córdoba, Spain; ^6^Montilla Hospital, Córdoba, Spain; ^7^Villarrubia Center, Occidente-Azahara Health Center, Córdoba, Spain; ^8^Faculty of Medicine and Nursing, University of Córdoba, Córdoba, Spain; ^9^Andalusian Health Service, Lucena Health Center, Córdoba, Spain; ^10^Andalusian Health Service, Sector Sur Health Center, Córdoba, Spain

**Keywords:** motivational intervention/interviewing, alcohol use, primary care, protocol, randomized controlled trial

## Abstract

**Background:**

Motivational interviewing (MI) could be a method for minimizing alcohol-related harm. The study aims to assess the effectiveness of a brief intervention, based on a MI, in patients with risky alcohol use attended in Primary Care (PC).

**Materials and methods:**

A cluster-randomized, two-arm parallel, multicenter, open-label, controlled clinical trial. Fifty PC healthcare professionals from the province of Córdoba (Spain) will be randomized to one of the two study groups: (1) Experimental Group (EG): MI-based approach; (2) Control Group (CG): Usual care based on health advice. EG intervention: Professionals will receive a training program focused on MI, consisting of a training workshop and the use of pre- and post-workshop questionnaires to measure knowledge and skills acquired, as well as the degree of empathy, with a videotape of the health professionals with standardized patients, before and after the workshop, and subsequent training feedback. CG intervention: Workshop on the management of risky alcohol use based on health advice; participants will also complete the pre-and post-workshop questionnaires and be videotaped. Study population: Patients ≥ 14 years old with risky alcohol consumption (28 Standard Drink Units-SDU-/week in men and 17 SDU/week in women) or excessive alcohol use (≥ 6 SDU in men or ≥ 4 SDU in women, in less than 2 h). It would be necessary to include 110 subjects/group to find a difference of 20% between the percentage of patients in abstinence between EG (37%) and CG (20%), alpha error of 5%, and statistical power of 80%. Assuming a loss rate of 5% and the cluster design effect, the number of subjects to be recruited is estimated at 197/group. The follow-up period will be 12 months. The primary outcome variables will be the self-reported alcohol use level and the Alcohol Use Disorders Identification Test (AUDIT) questionnaire score.

**Discussion:**

The study aims to demonstrate the effectiveness of the motivational approach in the comprehensive treatment of the patient with risky alcohol use, improving the empathy of the healthcare professionals and strengthening the healthcare professional-patient relationship to achieve the behavioral change of the patients with this problem in primary care consultations.

**Clinical trial registration:**

ClinicalTrials.gov.

## 1. Introduction

Alcohol consumption is one of the main causes of morbidity and mortality (1, 2). According to the World Health Organization (WHO), alcohol consumption caused approximately 3 million deaths worldwide in 2018 (5.3% of all deaths) and 132.6 million disability-adjusted life years (DALYs) (3). In the latest Global Status Report on alcohol and health published by the WHO, it is also mentioned that mortality resulting from alcohol consumption is higher than that caused by diseases such as tuberculosis, HIV/AIDS and diabetes. 1.2 million deaths from digestive and cardiovascular diseases (0.6 million for each condition) and 0.4 million deaths from cancers are related to harmful use of alcohol. Furthermore, risky alcohol use leads to disorders that cause family problems (such as mistreatment, unplanned pregnancy, separation) and economic and social consequences (unemployment, accidents, violence, homicides), and complicates the evaluation and treatment of other medical and psychiatric conditions (4).

In Spain, it is estimated that 4 million people have risky alcohol use and 2 million people meet criteria for alcohol dependence (5). According to the last Survey on Alcohol and Drugs in Spain (EDADES) (6), alcohol was responsible for 3.6% of deaths in 2017. Due to the individual and collective impact it causes, harmful alcohol consumption accounts for 15–20% of the consultations attended in Primary Care (PC). For this reason, PC healthcare professionals play a crucial role in the detection and care of patients with alcohol-related disorders by taking a comprehensive and personalized approach to lifestyle habits and toxic substance use (7).

In the GBD 2016 Alcohol Collaborators study published in August 2018 (8), in which a systematic analysis of alcohol use was performed in 195 countries from 1990 to 2016, it is concluded that alcohol consumption is an important risk factor for the global burden of disease and causes a substantial loss of health. It also determines that the risk of all-cause mortality, and specifically cancer, increases with higher levels of consumption, and that the level of consumption that minimizes loss of health is zero. In this study, an estimated 2.8 million deaths were attributed to alcohol consumption in 2016. Globally, alcohol consumption was classified as the seventh risk factor for premature death and disability in 2016. Among the population aged 15 to 49 years, alcohol consumption was the main risk factor for the overall burden of disease, causing 8.9% of DALYs (a useful measure for quantifying healthy life losses, either by premature mortality or by the time lived with reduced health) for men and 2.3% for women. In this same population, 3.8% of female deaths and 12.2% of male deaths were attributable to alcohol consumption. For populations aged 50 years and older, 27.1% of the total number of female deaths and 18.9% of male deaths were attributable to alcohol. In addition, the results of this study indicate that alcohol consumption and its harmful health effects could become a growing challenge. Therefore, it is crucial that alcohol control policies are enacted or maintained today to prevent the potential increase in alcohol consumption in the future (9).

Effective policies could now generate significant health benefits for the population in the coming years (10).

Despite the prevalence of this health problem, the intervention rates of PC health professionals in alcohol management are consistently low (11). In addition, a Cochrane review on the effectiveness of brief intervention in PC concludes that such brief interventions lead to a 12.3% reduction in the average alcohol consumption among those receiving it (12).

Similarly, it is also reflected in the literature that the implementation of motivational interviewing (MI) in PC is an effective communicational tool for addressing health problems (13–15), such as hypertension (16), dyslipidemia (17), or addictions, such as alcohol consumption (10). Although MI began to be applied in the management of risky alcohol use (10), there is little evidence about the actual effectiveness of MI in reducing alcohol-related harm in Spanish primary care centers. Given that the population assisted in primary care differs from the population assisted in the hospital setting, it is necessary to assess the effectiveness of a brief motivational intervention in the management of risky alcohol use in Spanish primary care centers.

The findings from the present study might explain the real benefits of the brief motivational intervention applied on patients with risky alcohol use, as well as making more informed health policy decisions when assigning potentially scarce resources.

## 2. Methods and analysis

### 2.1. Study design

**FIGURE 1:**
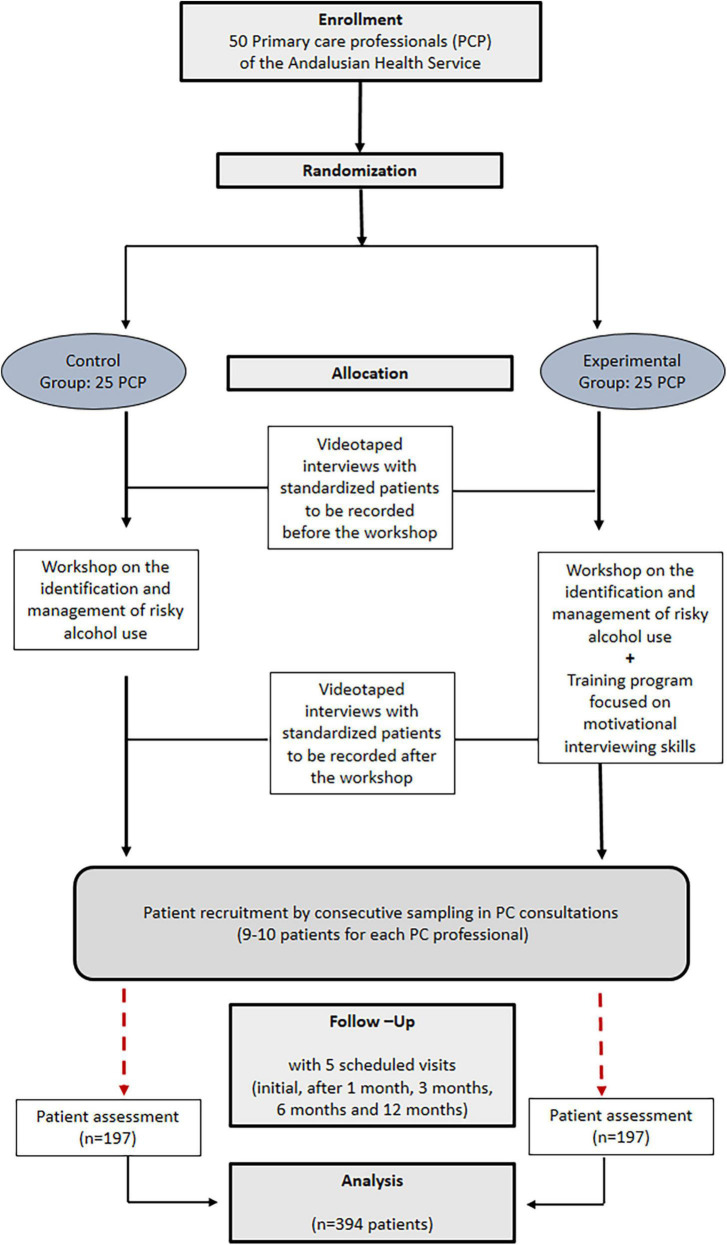
Cluster-randomized trial intervention scheme.

This is a cluster-randomized, two-arm parallel, multicenter, open-label, controlled clinical trial that will be conducted in PC centers of the Andalusian Health Service located in the province of Córdoba (Spain). PC professionals will be randomized to one of two study groups (Figure 1): (1) Experimental Group (EG): Professionals will implement an approach based on brief motivational intervention (18) applied on patients with risky alcohol use, receiving previously a specific training program in this field; (2) Control Group (CG): Professionals will also receive prior training on the management of the patient with risky alcohol consumption and will implement the usual clinical care (health advice) in recruited patients. Participants in both groups will attend a workshop focused on the identification and management of patients with risky alcohol consumption.

#### 2.1.1. Eligibility criteria

##### 2.1.1.1. Professional selection criteria

Inclusion criteria will be: (1) To be a PC healthcare professional (family physician, nurse or resident internal specialist in Family and Community Medicine or Nursing); (2) To provide informed consent to participate in the clinical trial.

Exclusion criteria will be: Prior MI skills or refusal to participate in the study.

At least 50 healthcare professionals will participate in the controlled clinical trial, each of them will recruit 7–8 patients in PC through opportunistic search.

##### 2.1.1.2. Patient selection criteria

Inclusion criteria will be:

1) To have risky alcohol use (19): (a) Consumption of more than 17 SDU–Standard Drink Units–of alcohol/week (170 grams of alcohol per week), for women; (b) Consumption of more than 28 SDU–Standard Drink Units–of alcohol/week (280 grams of alcohol per week), for men; (c) Patients with a score of 8 points or more in an AUDIT questionnaire; (d) Patients with “binge drinking” (excessive or intensive consumption). That is, males consuming 6 SDU or more or females consuming 4 SDU or more in less than 2 h.
2) To be at least 14 years old.
3) To provide informed consent to participate in the clinical trial.

Exclusion criteria will be:

1) Severe cognitive impairment (such as severe dementia or psychosis) and/or terminal illness.
2) Lack of social support or unemployment.
3) Coexistence of another drug dependency supervised by professionals specialized in addictions.

#### 2.1.2. Recruitment

The study will be disseminated through the Multi-professional Teaching Unit of Family and Community Care in the Health District of Córdoba and Guadalquivir. It is intended to recruit at least 15 PC physicians, a minimum of 15 PC nurses, and at least 20 resident physicians and internal nurses from the last year of training. All study information will be sent to the e-mail addresses of existing lists, teaching sessions or face-to-face meetings. Once the objective of the study has been explained, professionals will be invited to participate in the clinical trial and will complete the informed consent forms.

#### 2.1.3. Random assignment

The randomization unit will be the healthcare professional, and the intervention unit will be the patient. Professionals will be randomly assigned and equally (1:1) to one of the two study groups (EG or CG), stratifying according to the center and type of professional. Patients will be recruited through consecutive sampling (opportunistic search of subjects who are treated at participating health centers).

#### 2.1.4. Intervention planning

Before the intervention, participants will undergo the following training scheme:

-EG and CG will receive a 1-h workshop on the identification, and management of the patient with risky alcohol consumption, based on the recommendations and the algorithm of action proposed by the Program of Preventive Activities and Health Promotion-PAPPS-(20).

-EG: They will receive a 5-h training program to acquire specific MI skills for the management of patients with risky alcohol consumption, which will consist of a workshop, with two video recordings of consultations with simulated standardized patients, one prior to the training and the other after it. Finally, each participant will receive personalized feedback of the video recording from an expert. This program was accredited by the Andalusian Health Quality Agency (ACSA).

-CG: They will not receive the MI training program, instructing them only to perform the health advice they usually do with these patients (based on an informative-persuasive model). They will also be videotaped before and after the workshop to assess its formative impact and that the approach they perform does not present typical characteristics of MI.

The simulated standardized interviews will be conducted by two subjects (one male and one female, both middle aged), with previous experience in performing as actors. Using a role-playing technique, the actors will follow two scripts prepared by two team researchers, experts in the field, and will receive the timely formative feedback before the standardized interviews.

### 2.2. Materials and methods

Participants’ information will be obtained from validated tools for assessing risky alcohol use (AUDIT) (21) and the motivational interview (EVEM questionnaire) (22). In addition, the Jefferson (23) scale will be used to assess the empathy of healthcare providers in the management of alcohol use.

-AUDIT (21). Tool designed to identify risky alcohol use that comprises 10 questions divided into 3 conceptual domains. The first domain evaluates recent alcohol consumption and contains three questions (frequency of alcohol use, usual amount of alcohol consumption, and frequency of binge drinking). The second domain assesses symptoms of dependence through three items (loss of control over consumption, increased relevance of consumption and morning drinking). The third domain assesses harmful alcohol use through four questions (feeling of guilt after drinking, memory gaps, alcoholrelated injuries and concerns about drinking). A result equal to or above eight is considered indicative of hazardous and harmful consumption, and a possible alcohol dependence.

-Scale for the assessment of the MI (EVEM) (22). A scale of 14 items with a score of 0 to 4, created to assess encounters between professionals and patients using MI. This scale analyzes: (1) empathy; (2) facilitating patient positioning; (3) working in concordance with the patient; (4) using open questions; (5) performing reflective listening; (6) performing summaries; (7) validating the patient; (8) agreeing on change objectives; (9) promoting action/plan design with the patient; (10) Prevents discord with patient; Global Interview Spirit: (11) evokes; (12) collaborates; (13) honors patient autonomy; (14) shows compassion. This scale has been validated by members of this group (24).

-Empathy Scale (Jefferson Scale) (23), which evaluates three dimensions of empathy: Taking perspective, caring with compassion, and standing in the patient’s shoes. It consists of 20 Likert-type questions with a 7-point response range from strongly disagree (score = 1) to strongly agree (score = 7).

-Knowledge and Attitude Questionnaire: Based on the questionnaire created by our team for a previous study (25), and which was subjected to a process of apparent or consensus logical validity and content validity.

#### 2.2.1. Follow-up period

The follow-up period for each patient will be 12 months, with 5 scheduled visits (initial, after 1, 3, 6, and 12 months).

#### 2.2.2. Data collection and management

Measurements will be obtained as follows: (1) Pre-intervention: Initial or baseline data collection; (2) Intervention: Data will be collected at 1, 3, and 6 months after the baseline visit; (3) Postintervention: twelve months after recruitment, the patient will be interviewed to assess whether he/she maintains his/her status regarding the change of behavior over time.

The data obtained will be recorded in the data collection notebooks and sent online to the study coordinator for further processing, cleaning, and statistical analysis.

### 2.3. Statistical analysis

An intention-to-treat analysis will be performed to control the effects of losses and dropouts, dragging for analysis the data from the last observation obtained. Survey data will be automatically processed on Google Drive, directly by each of the participating researchers. They will then be exported to an Excel sheet from Google Drive and statistically treated with the SPSS v. 17.0. A descriptive analysis and an initial comparability analysis of the groups will be conducted. Confidence intervals of 95% will be calculated for the major study estimators. A bivariate analysis will be conducted to assess the relationship of the independent variables and the effect of the intervention, for which the Chi-square test will be used, the mean comparison test for independent samples, such as Student *t* test or ANOVA (after verification of normality by using the Kolmogorov-Smirnov test); bilateral comparisons were used, and a value of *p* ≤ 0.05 will be considered. A multivariate analysis will then be performed to determine which sociodemographic, work, and care factors are associated with the intervention developed, controlling the predictors and/or confounders by multiple linear regression and unconditioned binary logistic regression.

### 2.4. Sample size

Based on a previous study performed by our team members (26), and to find a 20% difference between the percentage of patients in abstinence (partial or total) between EG (37%) and CG (20%), for an alpha error of 5%, and statistical power of 80%, the size would be 220 subjects (110/group). Since it is a cluster randomization system, we will consider the “design effect” and we will assume a loss rate of 5%. Estimates of the intra-cluster correlation coefficient (ICC) in ECC by clusters in PC show that they are generally less than 0.05 (27). This ICC translates, for a cluster size of 15, into a design effect corresponding to a factor of 1.7. Assuming this value, the size would be 394 subjects to recruit (197 in each group).

### 2.5. Outcome measurements

#### 2.5.1. Dependent variables (endpoint or outcome)

**-Variables measured in healthcare professionals:**

The following variables will be measured to assess the impact of the training program on participants:

Primary outcomes will be the MI evaluation based on the EVEM scale (22) the patient-healthcare professional relationship based on the CICAA scale (28), and the professional awareness of alcohol use and attitude toward its approach.

Secondary outcomes will be the professional empathy based on Jefferson scale (23), and previous training of healthcare providers in MI.

**-Variables measured in patients with risky alcohol use:**

To assess the effectiveness of interventions, the main outcome variables will be the number of SDU in a typical day, the frequency of more than 6 SDU/day, and the total score of the AUDIT (21) questionnaire. All these variables will be considered as primary outcomes.

#### 2.5.2. Independent variables

**-Variables measured in healthcare professionals:**

The following variables will be recorded about the healthcare professionals: age, sex, occupation, supervisor of residents, time worked, place of work, contract type.

**-Variables measured in patients with risky alcohol use:**

The following variables will be measured in patients with risky alcohol use: age, sex, previous interventions, marital status, education level, place of residence, weight, height, body mass index (BMI), associated diseases, hygiene-dietary habits (such as smoking–number of cigarettes per day- and coffee consumption–number of coffees per day), and stage of change according to Prochaska and DiClemente’s Transtheoretical Model (18). We will also ask participants their opinion about their own alcohol consumption and its consequences in health through an open-ended question. Table 1 shows the variables that will be measured in participants at each follow-up visit.

**TABLE 1:** Variables to be measured at each follow-up visit.

Variables	Follow-up visits				
	**Baseline visit**	**1-month visit**	**3-month visit**	**6-month visit**	**12-month visit**
Socio-demographic characteristics	x				
Clinical measures: weight, height, body mass index (BMI), associated diseases, hygienic-dietary habits (smoking and coffee consumption).	x				
Alcohol consumption opinion	x	x	x	x	x
Standard drink units (SDU)/day of alcohol consumption	x	x	x	x	x
AUDIT questionnaire	x		X	x	x
Scale for the assessment of the motivational interview (EVEM)	x		x	x	
Stage of change regarding alcohol consumption	x	x	x	x	x

#### 2.5.3. Intervention evaluation

Each participant from the EG will complete a self-assessment after each visit using the EVEM questionnaire. Intermediate analysis of the results of these questionnaires will be performed to assess whether it is appropriate to perform any reinforcement training activities to try to improve the skills and abilities of EG participants in MI.

In addition, a video will be recorded with an actual patient at a randomly selected visit. Then, the expert evaluators will assess the healthcare professional skills using the EVEM scale.

## 3. Discussion

The present study aims to demonstrate that communication tools, such as MI, increase the effectiveness in managing patients with risky alcohol use in PC consultations. In addition, these tools strengthen the patient-healthcare professional relationship and allow, in turn, to improve the patient’s perception of the care received, helping us reduce the prevalence of this health problem. Likewise, the present study aims to promote preventive activities and enhance health education on such an important risk factor as alcohol consumption in PC, whose effect has been demonstrated by this research group (29, 30), and to provide continuity of care for this important public health problem. Similarly, the research study will help foster knowledge and communication skills and preventive recommendations of PC healthcare professionals, as well as provide support tools to facilitate decision-making aimed at reducing alcohol consumption in the general population.

The research project will allow patients with risky alcohol use suffering from a chronic, progressive and disabling disorder to be more effectively and comprehensively treated, since in most cases not even the PC health professionals themselves are sufficiently sensitized nor trained in these coping skills. Therefore, these patients are left out of the health system, with a progressive deterioration at the personal, social, occupational, and functional levels, which ends up generating an increase in the frequency of consultations due to other reasons, other than its dependence, but as a direct consequence of it, in addition to a greater risk of other chronic diseases. For this reason, the study will reduce the great social and also economic impact on the health system, resulting from the costs generated by these patients, both directly because of their excessive consumption of alcohol, as indirectly by the costs generated as a result of the innumerable comorbidities secondary to this risky consumption. This health savings would help reduce waiting times in consultation and provide higher quality of care in PC.

### 3.1. Strengths and limitations

#### 3.1.1. Strengths

It is one of the first research studies with a randomized, two-arm, comparative clinical trial design that assesses the impact of MI under real clinical conditions (effectiveness rather than efficacy study) in the field of PC in patients with risky alcohol consumption, giving it a more practical character and immediate clinical applicability, as its external validity increases. In the current literature, there are studies focused on risky alcohol consumption and its health impact (12), which assess the efficacy (i.e., under ideal or “laboratory” conditions) of MI. But none designed to compare the brief motivational intervention delivered by an EG and the health advice based on the PAPPS recommendations provided by a CG, in the framework of daily consultations in PC.

At the same time, the study may show whether or not there are differences in outcome between two types of interventions: MI-based versus most commonly used (simple health advice) in this type of problem where a change in behavior or health habit is attempted.

The approach is focused on MI as an intervention on the patient with risky alcohol consumption, not only in the preventive phase, but throughout the whole process (the patient is not going to be only a passive subject receiving an intervention, but an active person–empowered–able to decide/perform therapeutic activities to prevent/treat their dependence with the aim of reducing their consumption or achieving abstinence).

The present study will quantify the level of effectiveness of the therapeutic intervention as a percentage of successes (people who reduce their consumption/achieve abstinence), an aspect that is poorly evaluated and which greatly affects the achievement and maintenance of proposed functional objectives and patient satisfaction with the healthcare professional-patient relationship presented throughout the management of risky alcohol consumption, this has not been evaluated to date for this health problem.

#### 3.1.2. Limitations

It is necessary to keep in mind and recognize the possible bias known as the Hawthorne effect (observer bias) (31), which is inevitable or difficult to minimize in this type of experimental study, in which subjects usually change their behavior by the fact that they feel observed. If there is a high rate of professional or patient dropouts, withdrawals, or losses, the selection bias may become so important that can alter the actual results. Biases of information may occur due to the possible lack of sincerity of the respondent in answering questions regarding knowledge and attitudes regarding their alcohol consumption. Confusion biases will be controlled by multivariate analysis.

## Ethics statement

The studies involving human participants were reviewed and approved by Comité de Ética e Investigación Clínica del Hospital Reina Sofía de Córdoba (Spain). Written informed consent to participate in this study was provided by the participants’ legal guardian/next of kin.

## Author contributions

JF-G, JP-R, and LP-dT conceived the study. ER-R, LP-dT, GC-G, JP-R, and JF-G participated in the design of the study. AR-V contributed to the implementation. LP-dT provided methodological and statistical experience in the design of the clinical trial. JP-R and JF-G developed the scripts for the standardized patients. SF-L, AR-V, FL-C, and RF-M assisted in monitoring the videotaped interviews. All the authors read and approved the final manuscript.
