# Supplementary material for: Effectiveness of a brief motivational intervention in the management of risky alcohol use in primary care: ALCO-AP20 study protocol
Source: Front Med (Lausanne). 2023 Jan 13;9:1008832. doi: 10.3389/fmed.2022.1008832 (PMC9880185; doi:10.3389/fmed.2022.1008832)
Supplement: Supplementary file 1 [file Data_Sheet_1.PDF]

ALCO-AP Collaborative Group Study

Alejandro Camacho
Alejandro Girón Linares
Alicia Blanco Negredo
Alicia Moscoso Jara
Alicia Valenzuela Gómez
Ana García Vélez
Ana González de la Rubia
Ana Llera Morales
Ana Morilla Roldán
Ana Roldan Villalobos
Antonia Carmona Priego
Antonia Toledano Medina
Antonio León Dugo
Azahara García Gallego
Carmen Jurado Porcuna
Carmen Rodríguez Buza
Carmen Sánchez Aguilar
Celia Pérula Jiménez
Cristian Brata
Cristina Rojas Prats
Cristina Ruiz Rull
Daniel Cabello Morales
Elena María De Dios González
Elena Ruiz Rojano
Enrique Martínez Martínez
Esperanza Romero Rodríguez
Estrella Castro Martín
Eva María Sánchez Cañete
Fatima Bravo Abalos
Fernando González Martínez
Francisco López Cañas
Gertrudis Montes Redondo
Helena Cruz Terrón

Inés Gutiérrez París
Isabel Jabato Moreno
Javier Cazalilla Expósito
Jesús González Lama
Jesús Villar García
Jose Luis Zambrana Luque
José Tomás Linares
Juan Baleato Gómez
Juan José León Serrano
Juan Marcos Baños
Julia Hervás Jerez
Laura Aranda Domínguez
Laura Martín Guerra
Manuel Marín
Manuela Urbano Priego
Margarita Fernández Poyatos
María Angeles Quesada Román
María Bello Castro
María Carmen Márquez Córdoba
María Carmen Ocaña Rodríguez
María del Carmen Castillo
María del Carmen Luna Moreno
Maria del Carmen Membiela Jurado
María Dolores López Espejo
María Dolores Ramos
María Isabel López Estepa
María Luisa Soria Cabrera
María Luisa Trigueros Guerra
María Reyes Martínez Guillén
María Sierra Henares
Marina Guijarro Blanco
Marta Alba Priego
Marta Espejo Marín
Marta Lomas Alarcón
Miguel Muñoz Álamo

Nazaret María Vargas Berni
Nazaret Morales Delgado
Raquel Aguilera Muñoz
Raquel Gracia Rodríguez
Raquel Sauces Carrillo
Rocío Luna Cuevas
Rodrigo Fernández Márquez
Rodrigo Ruz Muriel
Rodrigo Sebastian Fernández Márquez
Rosalía Serrano Berni
Samuel Postigo Farrabal
Santiago Gascón Veguín
Sharon Stefany Marín González
Sofía Chico Tierno
Tránsito Porras Castro
